# Parliament and the Profession

**Published:** 1916-02-05

**Authors:** 


### PARLIAMENT AND THE PROFESSION.

### Nurses and their Passports.

Mr. Watt questioned the Under-Secretary for War with regard to the fact that nurses in uniform passing backward and forward to France are not granted the same privileges as soldiers, or any advantages over the general public, in having their passes examined. Mr. Tennant replied that nurses in the uniform of the Q.A.I.M.N.S. and others who travel with military warrant embark and disembark from military gangways, and are on the same footing as soldiers. Ladies temporarily employed as nurses with certain voluntary organisations who do not travel on military warrants or under military orders pass the examination of passports as all passengers do. The regulations to this effect were drawn up by the French and British military authorities, and are necessary in order to prevent impersonation.

### Soldiers and the Selection of Hospitals.

The desirability of sending sick and wounded soldiers to hospitals as near as possible to their own homes was urged by Mr. Barnes, who, in a question addressed to the Financial Secretary to the War Office, pointed out that it was inconvenient to the relatives of soldiers who had to undergo treatment at some place far distant from home. In his reply, Mr. Forster said it was not possible to send every man to the hospital to which he would prefer to be admitted, but arrangements could generally be made for the patient's transfer to a hospital near his home as soon as he was fit to travel.

### Medical Appliances in Military Hospitals.

Mr. Gwynne suggested, in a question he asked the Under-Secretary for War, that certain medical appliances required by military hospitals had to be ordered from Woolwich, often entailing a delay of several weeks. Mr. Tennant, in his reply, said the regulations provided for local purchases under specially urgent circumstances not admitting of delay, but as these local purchases were a more expensive method of meeting requirements than obtaining what was wanted from War Office contractors, they were restricted to cases of urgent necessity.

### Other Matters of Interest.

Income Tax and Officers of the R.A.M.C.—Replying to Mr. Watt, Mr. Montagu said that no general notice with regard to income tax had been issued to officers of the R.A.M.C., but that the proper rate of deduction from the pay of officers whose total income exceeds £300 and does not exceed £1,000 was 1s. 9¾d. in the £.

The Pay of R.A.M.C. Officers.—Replying to a question asked by Mr. Laurence Hardy, Mr. Forster stated that captains in the Royal Army Medical Corps Special Reserve receive the same pay as captains in the corresponding corps in the Regular Army, and that their emoluments are nearly the same as those of temporary lieutenants or captains serving under civil contract.

Medical and Dental Officers' Rations.—Replying to Sir C. Kinloch-Cooke, Mr. Forster stated that the contract in respect to rations in the case of temporary medical officers was the same as in the case of dental officers while in the field; both contracts provide for the issue of free rations.

